# Exploring Healthcare Providers’ and Women’s Perspectives of Labor Companionship during Childbirth: An Interpretative Phenomenological Analysis Study

**DOI:** 10.3390/healthcare12090869

**Published:** 2024-04-23

**Authors:** Anwar Nader AlKhunaizi, Areej Ghalib Al-Otaibi, Manal F. Alharbi, Ghareeb Bahari

**Affiliations:** 1 Maternal and Child Health Nursing Department, College of Nursing, King Saud University, Riyadh 11421, Saudi Arabia; aalkhunaizi@ksu.edu.sa (A.N.A.); maalwahbi@ksu.edu.sa (M.F.A.); 2 Fundamental of Nursing Department, College of Nursing, Imam Abdulrahman Bin Faisal University, Dammam 34212, Saudi Arabia; agalotiaby@iau.edu.sa; 3 Nursing Administration and Education Department, College of Nursing, King Saud University, Riyadh 11421, Saudi Arabia

**Keywords:** benefits, birth, challenges, companionship, healthcare providers, mothers

## Abstract

A labor companion of choice during childbirth is crucial for improving women’s birth experience and confidence to give birth. Labor companions provide various benefits, including enhanced communication, emotional support, non-pharmacological pain relief, and better healthcare. However, little is known about the supportive actions of labor companions with respect to women’s needs during labor and birth, as well as healthcare providers’ perceptions of labor companions. Therefore, this study was conducted to explore the perceptions of healthcare providers and women regarding labor companions. The study utilized an interpretative phenomenology research design. Data collection involved conducting semi-structured interviews with 14 participants. The sample consisted of mothers, physicians, and nurses, ensuring a diverse range of perspectives. An interpretative phenomenological analysis was conducted for data analysis. Five themes were identified: (a) impact of companionship, (b) benefits for healthcare providers, (c) companion roles, (d) loneliness and alienation of mothers, and (e) challenges of implementation. The findings indicated that the presence of a companion reduces the need for unnecessary medical interventions and eases the workload of healthcare providers. Without a companion, mothers often feel lonely and disconnected during the birthing process. The presence of companions is often hindered by space limitations in delivery rooms, the absence of clear policies, and lack of childbirth education programs for companions. Clear policies, education programs, and adequate space are essential for implementing and promoting labor companionship during childbirth.

## 1. Introduction

Improving the quality of care during childbirth is a significant concern internationally [1]. Having a companion of choice during childbirth is an important component of good quality and respectful maternity care and is standard practice in many countries [2]. Labor companions can help improve communication and understanding between women, families, and healthcare providers, facilitate non-pharmacological pain relief such as massages, mobility assistance, and position changes, and provide women with crucial emotional support and reassurance [3]. These benefits to women’s health and wellbeing can ultimately improve birth experiences and build women’s confidence in their ability to give birth [4]. Moreover, women who do not have a labor companion are more likely to experience physical abuse, not feel in control, and struggle with poor communication [5].

However, barriers to the implementation of labor companionship include limited education for women, families, and healthcare providers on the benefits of companionship, restrictive policies at the health facility level, and limited space or privacy in health facilities [3]. Rules for labor companionship have not yet been established in many countries, and many healthcare facilities do not permit women to have a companion. Thus, guidelines regarding labor companionship differ across hospitals [3,6].

Saudi healthcare transformation programs draw a roadmap toward providing high-quality healthcare services. This transformation is included in one of the strategic goals of Saudi Vision 2030, which has three main pillars: a vibrant society, a thriving economy, and an ambitious nation. One transformative goal is to improve healthcare by improving the quality and consistency of services to deliver safe, effective, and equitable care. Maternity care benefits are transformational services that seek to achieve this vision by helping women have safe deliveries and healthy infants in Saudi Arabia [7].

During labor and childbirth, emotional and social support can be provided to women by a companion of choice, such as a husband or family member [4]. When a woman has access to a trusted source of emotional, psychological, and practical support during labor and childbirth, her childbirth experience and health outcomes could improve. The WHO recommendations on intrapartum care for a positive childbirth experience recommend a labor companion of choice for all women giving birth. As with other nonclinical interventions, labor companionship has not been prioritized in many settings. Nevertheless, it is an essential component of the care experience [8].

The presence of a companion provides laboring women with an enhanced feeling of control and reassurance, increases their satisfaction, decreases their anxiety and perceived pain [9], and may have a positive influence on staff treatment and interactions with the women [10]. Moreover, a stressful and unfamiliar environment activates the sympathetic nervous system and inhibits the release of endogenous oxytocin, leading to weakened or stopped labor contractions, whereas a calm, private, and safe environment reduces stress and increases the release of endogenous oxytocin [11].

Many programs have been created to improve women’s healthcare status during labor. However, research on healthcare providers’ and women’s perceptions regarding labor companions is lacking. Therefore, this study was conducted to explore healthcare providers’ and women’s perspectives on labor companionship during childbirth. The findings of this study may facilitate the development of a training model for companions to clarify their roles. Moreover, they can inform policy decisions, improve women’s birthing experiences, and promote patient-centered care during childbirth.

## 2. Materials and Methods

### 2.1. Study Design and Setting

The present study utilized an interpretative phenomenology research design to explore the perceptions of healthcare providers and women regarding labor companions. The participants were selected from a governmental hospital in Eastern Province, Saudi Arabia. The hospital, which has a 375-bed capacity and serves approximately one thousand mothers visiting the antenatal clinic, is considered the largest in the area. It is recognized as a mother-friendly hospital as it provides maternal care services with high standards and quality [12].

### 2.2. Sampling Process

Purposive sampling was employed in this study to recruit participants. The study consisted of three groups: four mothers, six physicians (including obstetricians and gynecologists), and four nurses. The data saturation approach was used to determine the required sample size; data collection was stopped when additional participants no longer provided further insight [13]. The inclusion criteria included Saudi women with English fluency to understand the interview questions. Though the official spoken language in Saudi Arabia is Arabic, the interview was conducted in English as it is the official language of nursing and hospitals in the country. The research team ensured diversity among the women in terms of parity, age, and obstetric history (e.g., with or without a previous cesarean section). The healthcare providers were required to have a minimum of five years of experience in maternity units. These criteria aimed to capture diverse and comprehensive lived experiences related to labor companions.

### 2.3. Data Collection Procedures

A study announcement was posted on bulletin boards at the hospital and sent by email to the target population. Additionally, the word-of-mouth strategy was utilized to facilitate participant recruitment. The interviews were conducted in November 2023. Each participant was individually interviewed to ensure their privacy and the confidentiality of their information. All interviews were semi-structured, and the researcher compiled a list of questions and points to cover (Table 1). The interview sessions ranged in length from approximately 40 to 60 min and were conducted in a convenient place. The researchers utilized audio recording equipment to document participants’ responses. Questions based on participants’ views, such as “Why?” “How?” or “What do you mean by this?” were asked to obtain further helpful information.

### 2.4. Study Trustworthiness

To determine the quality of the collected qualitative data, four methods of including credibility, dependability, confirmability, and transferability were ensured [14]. The researchers conferred with the interviewees and requested feedback on the transcripts to ensure credibility. Dependability was ensured through sharing the findings with healthcare providers and mothers not involved in this study for verification. To ensure confirmability in this study, participants were asked to verify that the narratives accurately reflected their true feelings and opinions. Finally, to assess transferability, we determined whether the results can be applied to similar populations, such as in other regions in Saudi Arabia. Employing these methods helped ensure the rigor of the findings.

### 2.5. Ethical Considerations

This study was approved by the institutional review board of Qatif Central Hospital (Reference # QCH-SRECO 52/2023), dated 17 September 2023. The participants were informed about the study procedure, asked to participate voluntarily, and reminded of their right to withdraw at any time without any penalty. They were assured that their responses would be kept confidential and only accessible to the members of the research team. To maintain confidentiality, participants were assigned codes to use instead of their names. Finally, all participants agreed to be recorded, and they were assured that all responses and transcripts would be securely destroyed. Informed consent was obtained from all participants.

### 2.6. Data Analysis

All interviews were audio recorded and transcribed verbatim. To collect detailed and unbiased feedback from the participants, the interviews were guided by research questions. The data from each participant were analyzed in an iterative manner, allowing for revisions to be made based on previous interpretations. Once the interviews were conducted, recorded, and transcribed, an interpretative phenomenological analysis was conducted [15]. This method focused on revealing the participants’ viewpoints and gaining a deeper understanding of their experiences through an idiographic and inductive approach. The idiographic approach is linked to understanding specific cases and unique individuals [16]. The steps of interpretative phenomenological analysis are listed in Table 2. An expert in qualitative studies was also asked to review and confirm the analysis process and findings.

## 3. Results

### 3.1. Sample Characteristics

Six physicians, four nurses, and four mothers from the obstetrics and gynecology departments provided their consent to participate in the interview sessions.

### 3.2. Findings from the Semi-Structured Interviews

The qualitative findings were related to five themes regarding companions for women in labor. The themes are as follows: 1. impact of companionship, 2. benefits for healthcare providers, 3. companion roles, 4. loneliness and alienation of mothers, and 5. challenges of implementation.

#### 3.2.1. Impact of Companionship

This emerging theme was developed based on the perceptions of healthcare providers and women regarding the presence of a labor companion. The labor companion’s role is to provide physical and mental support to the mother throughout the labor and birth process, giving her the strength to undergo the challenging journey. This, in turn, can help to reduce the need for unnecessary medical interventions and contribute to a positive and satisfactory experience. Example comments are provided as follows:

“It is crucial that nurses support mothers during the labor and delivery process by allowing a companion to be in the delivery room. This calms the mother, gives her strength and enables her to feel safe. Whilst working as a nurse, I have learned that having a companion is vital in ensuring the mother’s safety and support.”

(Nurse AM)

“The presence of someone supportive during labor will reduce feelings of stress and fatigue for the mother, which ultimately improves her birthing experience. The ability for family members, such as a spouse or sister, to be with the mother throughout labor improves the mother’s birthing experience.”

(Physician D)

“A companion during labor and delivery is a source of reassurance, comfort and support to the mother. A companion can make the mother feel safe and less alone, allay her fears and reduce her pain.”

(Physician FA)

Two participants explained the positive impact on breastfeeding, as follows:

“Primigravida mothers will benefit from the presence of a companion, who will support them in breastfeeding their newborn and encourage skin-to-skin contact.”

(Nurse Z)

“In doing so, they can support her during childbirth by easing her pain and making the experience enjoyable. This also reduces the mother’s postpartum discomfort and improves both skin-to-skin contact and breastfeeding.”

(Physician R)

#### 3.2.2. Benefits for Healthcare Providers

We found that the presence of a companion is advantageous not only for mothers but also for healthcare providers. The healthcare providers indicated that the presence of a companion decreased their workload. Example responses are:

“As a consultant in obstetrics and gynecology, it is my responsibility to encourage the mother to have a companion throughout labor and delivery, as this enhances the experience of giving birth. The woman cooperates more with us as healthcare professionals and the nurses work less during the labor process as the mother is calm and cooperative. This results in a positive delivery experience.”

(Physician D)

“The companion’s presence thus benefits not only the mother, but also the healthcare provider. Given the fact that there are not enough nurses in delivery suites to ensure that each mother constantly has a nurse by her side, mothers are often left alone while the nurses focus on other patients. The companion’s presence helps the mother to keep calm and cope with pain at this point in the labor process.”

(Mother K)

#### 3.2.3. Companion Roles

This theme emerged based on healthcare providers’ and women’s perceptions regarding the roles of the companion in the delivery room, including helping with exercise, massaging, and facilitating breathing exercises. Some participants commented on the healthcare providers’ role to encourage the presence of the companion in the birthing room. Example responses are:

“The husband can also help her to change positions, do exercises during labor and use the birth ball. A companion’s presence will reduce the need for unnecessary medical interventions, such as giving the mother epidural anesthesia, analgesics or amniotomy. In addition, the companion will encourage the mother to breastfeed immediately after giving birth, in the golden hour.”

(Nurse AM)

“This is particularly true for primigravida mothers, who often experience fear. However, the companion must know how to calm the mother without resorting to screaming, but rather using breathing techniques. The companion supports the mother both physically and mentally. A companion must be there for the mothers throughout the labor and delivery process. Having a companion in the delivery room has a significant influence on the mother’s feelings during labor. However, the companion often feels a combination of sadness at the mother’s pain and joy that she has reached such a milestone in her life.”

(Nurse AM)

“A companion must be able to provide support through exercises, massages and assistance with breathing techniques.”

(Physician R)

“The companion is tasked with supporting the mother and providing encouragement during childbirth. This may include helping her to perform exercises, walk and use the birthing ball.”

(Physician RE)

#### 3.2.4. Loneliness and Alienation of Mothers

This theme describes that mothers were lonely without the presence of a companion. Mothers expressed concern about their feelings of alienation, particularly when they were left alone in the birthing room. Example responses are:

“I did not have a companion by my side in the delivery room, and went through labor and delivery on my own. This meant that I felt lonely and had to cope with pain without a familiar companion. Since the medical and nursing staff were strangers, I felt stressed and anxious rather than safe and psychologically and physically supported. The absence of a companion had a negative impact on my experience of childbirth and the levels of pain I experienced.”

(Mother FD)

“My personal experience of giving birth is one of fear, pain and stress, since I was left to cry and scream alone. My husband, mother and sisters did not come with me, so I had no one to turn to for psychological and physical support—precisely the role which companions fulfill. I hope that every hospital will recognize the importance of mothers having a companion by their side during labor and delivery.”

(Mother FI)

#### 3.2.5. Challenges of Implementation

This last theme explores the various challenges revealed in the interviews. These challenges include the limited space in the birthing room, the absence of single rooms in certain hospitals, the lack of clear hospital policies supporting the presence of companions, and the absence of a childbirth education program for companions. Participants discussed the problems stemming from insufficient space in the delivery room as well as education and training, which directly impact the ability of companions to be present. Some example responses are:

“There are a number of obstacles hindering the ability to have a companion present with the mother during labor and delivery, one of the most significant of which is the limited space in the delivery rooms of government hospitals, in stark contrast to private hospitals.”

(Nurse AM)

“There are several obstacles that can hinder this process, including the provision of childbirth education to couples wanting to have a natural childbirth. At present, training is insufficient for mothers and their husbands.”

(Physician R)

“One major obstacle is the fact that there are too few accredited programs for educating companions on the importance of helping mothers change position during labor. This knowledge and training are essential for ensuring the mother’s birth experience is positive and has a successful outcome.”

(Nurse FW)

“Among the obstacles to the introduction of labor companions in Saudi Arabian hospitals is the lack of available intensive courses on natural birth support in the hospital where the mother will give birth. Another obstacle is the potential lack of family support.“

(Nurse AM)

“There are no chairs for the companion to sit during the labor process. Additionally, the mother’s privacy is a primary concern. Couples require privacy during childbirth.”

(Physician RE)

The participants described the lack of clear policy from upper management and their negative impact on birth experience, as follows:

“The number of births at hospitals where a partner is not permitted to be present during labor and delivery has decreased as a result of this policy.”

(Physician D)

“There is no clear written policy on the role of the birth companion which would facilitate companions being able to attend labor and delivery.”

(Physician M)

## 4. Discussion

In this study, we used an interpretative phenomenology research design to explore healthcare providers’ and women’s perceptions of labor companions. The findings included a core category of supporting birth companions for women in labor with five themes: the impact of companionship, benefits for healthcare providers, companion roles, loneliness and alienation of mothers, and challenges to implementation of companionship during childbirth. The analysis yielded themes that provide insight into the importance of labor companionship for women in Saudi Arabia. The findings show that the participants were aware of the importance of the companion in supporting the mother physically and mentally during the labor and delivery. Some of the advantages provided by the companions included encouragement for the women, a reduction in the need for unnecessary medical interventions, and the promotion of satisfactory childbirth experiences.

Other studies have highlighted similar advantages. A study showed that the presence of a companion provides laboring women with a greater feeling of control and reassurance and decreases anxiety and perceived pain [9]. Similarly, continuous, one-to-one intrapartum support during labor could improve outcomes for women and infants, including spontaneous vaginal birth, shorter duration of labor, and lower likelihood of cesarean birth, instrumental vaginal birth, use of any analgesia, low five-minute Apgar score, and negative childbirth experience [17,18]. Furthermore, women’s and health workers’ perceptions of labor companionship in a public maternity unit in rural Kenya were also explored. Researchers identified similar advantages and impacts of labor companions as well as the roles they could play [17].

The presence of a companion during childbirth had a positive impact on breastfeeding, which is consistent with another study that showed a significant association between the presence of a birth companion and breastfeeding initiation [18]. However, these findings contradict another study that showed no apparent effect of companion support on breastfeeding [19]. Additionally, our findings indicated that healthcare providers can benefit from labor companions. Some participants commented on the provider’s responsibility to encourage the presence of the companion in the birthing room. Similarly, previous studies concluded that healthcare providers are the gatekeepers of companionship in organizations with staff shortages and work overload [2,10]. They also found that the introduction of labor companionship was well accepted by healthcare providers, government officials, and, most importantly, women who delivered at those healthcare organizations.

The concept of companion roles emerged as a key theme in our study. The participants considered these roles to involve activities such as helping the mothers to exercise, massaging, and facilitating breathing exercises. According to a study conducted by Bocoum and colleagues, the current function of a companion is to connect the birthing woman with healthcare professionals [14]. Moreover, mothers perceived a lack of companionship as a cause of loneliness and isolation [20]. Additionally, authors demonstrated that many participants experienced depression due to giving birth alone [21]. By contrast, having a companion present during labor and childbirth lessens the pressure on women to manage their pain and allows them to freely express their needs [14].

Furthermore, our research identified several challenges that arise in the implementation of companionship. These challenges encompassed issues such as limited space in delivery rooms, the absence of private rooms in certain hospitals, the lack of clear hospital policies supporting companion presence, and the absence of educational programs for companions. Similarly, a study conducted in Burkina Faso recognized the benefits of having labor companions but also highlighted obstacles to implementing this practice, including limited space, hospital regulations, and social norms [14]. These barriers are remarkably similar to those identified in a prior study conducted in public hospitals in Thailand, which concluded that limited physical space in labor wards and multiple beds in the same labor room were major obstacles to introducing labor companionship [8]. Similarly, many of the same barriers to allowing a companion of choice during labor in supply-strained hospital settings, including the crowding of shared labor rooms and the absence of clear communication with the companion about their role were identified [22].

Based on our research, it is crucial to offer social support and provide mothers with companionship during labor. Therefore, measures should be undertaken to ensure the implementation of this practice at the national level. Policymakers should concentrate their efforts on this concept. Furthermore, they should actively endorse the notion of granting mothers the autonomy to select their preferred companion. Individuals should be educated about the invaluable role of companions and the extent of freedom mothers should possess when making such decisions. Moreover, mothers, along with their chosen companions, should receive comprehensive guidance concerning the necessary protocols upon their arrival at the hospital. To ensure privacy and a comfortable environment for mothers, certain modifications to birthing rooms must be made. This may involve the incorporation of curtains and room partitions or the provision of a separate room for each mother. Ultimately, hospital policymakers must establish a mandate allowing mothers to be accompanied during normal birth and cesarean sections.

### 4.1. Study Limitations

Some participants expressed concerns about being recorded when sharing their thoughts. To resolve this, we confirmed that their data would be kept confidential and reviewed only by the research team members. Additionally, we confirmed that all data would be deleted upon full completion of the study. Further, as qualitative research relies on participants’ experiences and opinions and researchers’ interpretations and subjective judgments, some subjectivity and interpretation challenges may be introduced. Another limitation of the study was that only English-speaking women participated, which could limit the generalizability of the findings. Therefore, it is important for future research to include participants with diverse language backgrounds in order to provide valuable insight applicable to different settings. To achieve a more comprehensive understanding, future studies should also consider including women from various public and private hospitals and involving companions’ perceptions. Conducting research across different healthcare settings would provide further support for the benefits of labor companionship during childbirth.

### 4.2. Study Implications

Healthcare organizations rarely engage with and fully utilize birth companions in childbirth preparation, as these companions require orientation and preparation to offer psychosocial support. Involving both the mother and her birth companion in a childbirth preparation program will ensure the quality of prenatal care. Moreover, empowering mothers in terms of birth companionship may have a long-term positive impact on maternal social support within the community. Finally, implementing a companionship policy by policymakers will help to achieve higher levels of empowerment and autonomy for mothers.

## 5. Conclusions

This qualitative study explored the perceptions of mothers and healthcare providers regarding labor companionship. All participants demonstrated awareness of the advantages of companionship. The active participation of birth attendants and the provision of adequate support and resources can help to enhance the wellbeing of mothers and contribute to a positive experience during their hospital stay. However, additional efforts are required to develop and implement a clear policy across healthcare organizations, empowering mothers to make choices that lead to high-quality care. Such a step would support the positive effects of labor companionship and its wider adoption.

## Figures and Tables

**Table 1:** Interview Guide.

From your experience, what do you think are the benefits of implementing a labor companion? From your experience, what do you think are the advantages of having a companion? From your experience, what do you think are the barriers to the successful implementation of labor companions in Saudi Arabian hospitals? What are your feelings about being allowed or denied a companion during labor? From your experience, what do you think are the reasons for desiring a companion during labor? From your experience, what do you think are the reasons why companions sometimes may not be allowed in the ward? From your experience, what roles are performed by a companion during normal labor and childbirth? Please explain.

**Table 2:** Step by step to conducting IPA analysis.

Step 1: Starting with the first case: reading and rereading
Step 2: Explanatory notes
Step 3: Constructing experiential statements
Step 4: Searching for connections across experiential statements
Step 5: Naming the personal experiential themes and consolidate
Step 6: Continuing the individual analysis of other cases
Step 7: Working with personal experiential themes to develop group experiential themes across cases

## Data Availability

The data presented in this study are available upon request from the corresponding author due to privacy and ethical restrictions.
